# A multimodality imaging approach to the diagnosis of infective endocarditis: incremental value of combining modalities

**DOI:** 10.1007/s10554-025-03480-0

**Published:** 2025-08-18

**Authors:** Siobhan Boyle, Alexandra Arvanitaki, Kieran Oldfield, Zoi Tsoumani, Giulia Iannaccone, Polona Kacar, Arun Dahiya, Claudia Montanaro

**Affiliations:** 1 Department of Cardiology, Royal Brisbane and Women’s Hospital, Logan Hospital, Royal Brompton Hospital, University of Queensland and Griffith University, Brisbane, QLD 4066 Australia; 2 Aristotle University of Thessaloniki, Greece and Royal Brompton Hospital, London, UK; 3 The Prince Charles Hospital and Griffith University, Brisbane, Australia; 4 Royal Brompton Hospital, NHS Foundation Trust, Guy’s an St Thomas, London, UK; 5 Universita` Cattolica del Sacro Cuore and Fondazione Policlinico Universitario, Rome, Italy; 6 Royal Brompton Hospital, London, UK; 7 The Princess Alexandra Hospital, Logan Hospital, University of Queensland and Griffith University, Brisbane, Australia; 8 Royal Brompton Hospital and Imperial College London, Bambino Gesù Hospital Rome, Rome, Italia

**Keywords:** Infective endocarditis, Multimodality imaing in infective endocarditis, Cardiac CT in infective endocarditis, Cardiac PET in infective endocarditis, Cardiac MRI in infective endocarditis, Echocardiography in infective endocarditis

## Abstract

Infective endocarditis is a complex disease leading to significant morbidity and mortality. The in-hospital mortality has remained at 15–30% despite significant advances in cardiac imaging over the past two decades. Prevention, timely diagnosis, and prompt treatment can minimise adverse sequelae. The key to prevention lies in patient and clinician education. Traditionally the diagnosis has relied on the modified Duke criteria which have a sensitivity of 80%. The limitations of the modified Duke criteria are widely acknowledged, particularly where prosthetic material is present (i.e. in prosthetic valve replacements, intracardiac devices, aortic grafts and in congenital heart disease) and visibility with echocardiography alone may be poor. Advancements in multimodality imaging techniques in recent years offer additional information to improve the diagnostic process and optimise patient care. Multimodality imaging in the diagnostic work-up for suspected endocarditis now forms part of the major society guidelines. We present a case series and review of the literature on the use of multimodality cardiac imaging in the diagnosis of infective endocarditis with specific reference to transthoracic and transoesophageal echocardiography, cardiac computerised tomography (CT), cardiac positron emission tomography (PET) and cardiac magnetic resonance imaging (MRI) to illustrate the complimentary role of each modality.

**Graphical Abstract:**

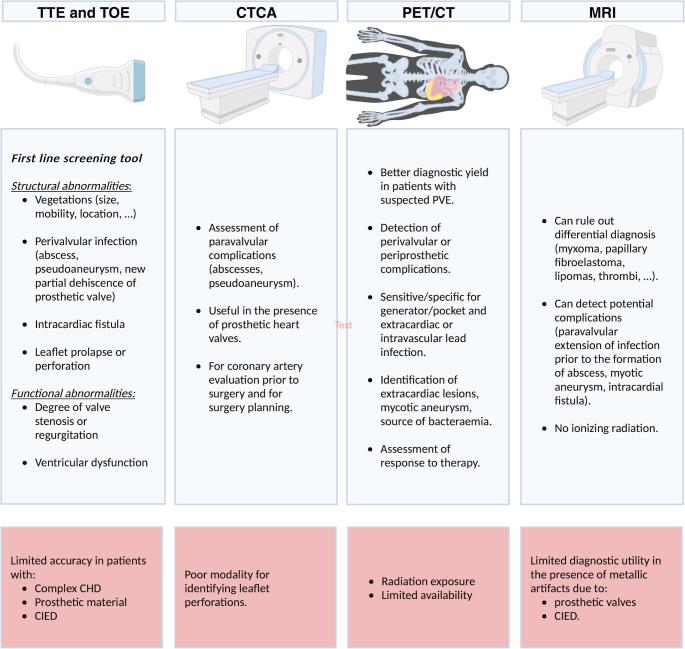

## Introduction

Despite advances in the diagnosis and treatment, infective endocarditis (IE) remains a major public health challenge [1]. Strategies for prevention are important to reduce mortality and morbidity, but the outcome of infective endocarditis depends mainly on timely recognition and appropriate treatment. Accurate, prompt diagnosis often proves difficult because of the diverse clinical manifestations [2, 3]. Echocardiography is the first-line imagining modality in patients with suspected IE which can detect the presence of IE as well as assess the functional and structural damage to cardiac structures [2, 4]. Its findings have prognostic implications and help with the decision-making process regarding medical or surgical treatment [2–5]. Echocardiography may be normal or inconclusive in up to 30%, [5] particularly when there is prosthetic material present or in cases of complex congenital heart defects [6, 7].

Other imaging modalities, such as nuclear imaging, computed tomography (CT), and magnetic resonance imaging (MRI) have been shown to be complementary in demonstrating valve involvement as well as endocarditis on distal structures [8–10]. A multimodality imaging approach was introduced in 2015 and modified in the current ESC guidelines and in the modified Duke criteria [1, 11].

We aim to describe the use of multimodality cardiac imaging in the diagnosis of infective endocarditis with specific reference to transthoracic and transoesophageal echocardiography, cardiac computed tomography, cardiac positron emission tomography and cardiac magnetic resonance imaging, using several cases from our institution to illustrate the complimentary role of each modality.

### Prevention: clinician and patient education

The high risk of adverse events associated with IE makes it critical to optimize preventive strategies. Clinicians should identify patients at elevated risk. Current guidelines indicate that patients with previous IE, prosthetic heart valves or with any material used for cardiac valve repair or congenital heart disease (CHD) are at increased risk of IE. Patients with rheumatic and non-rheumatic heart valve disease, CHD with isolated valvulopathy, cardiac intravenous electronic devices (CIED) and hypertrophic cardiomyopathy, instead, are considered at intermediate risk. Additional extracardiac factors, such as extremes of age and immunocompromised states, may increase predisposition to IE [1].

Healthcare providers should provide individuals at the highest risk with personalized infection prevention counselling and support. Patient education is the cornerstone of prevention [12]. The relevance of professional regular oral care and attentiveness to skin hygiene (i.e. wound care) should be highlighted. Those at intermediate and high risk should be discouraged from getting tattoos and piercings [1]. Most importantly, patients should be educated about IE signs and symptoms and advised to seek urgent medical attention and early antimicrobial therapy in case of possible or confirmed infection [1–3].

Appropriate risk stratification is recommended but remains challenging. It has been widely demonstrated that unregulated antibiotic prescription leads to the development of multi-resistant bacterial species which are difficult to eradicate. [13] Antibiotics have side effects and can have serious adverse effects for individuals, such as anaphylaxis. At present, antibiotic prophylaxis is recommended by the ESC in patients considered at high-risk of endocarditis (as outlined above) in patients undergoing ‘at risk’ dental procedures including dental extractions, oral surgery or procedures requiring manipulation of the gingival or periapical region of the teeth [1]. The recommendations from the American Heart Association and American Dental Association are very similar [14]. Whilst recommendations and guidelines may vary, prophylaxis may also be considered in intermediate risk individuals and an individualized assessment is advised [1]. Prophylaxis may be considered in high-risk patients undergoing invasive procedures of the respiratory, gastrointestinal, genitourinary, skin, or musculoskeletal systems [1]. For cardiac procedures, pre-operative screening for nasal carriage of *Staphylococcus Aureus* and subsequent treatment is recommended before elective cardiac surgery or transcatheter valve implantation [15]. Exclusion of potential sources of sepsis should be considered two weeks prior to cardiac procedures where possible (i.e. in elective cases). Peri-operative antibiotic prophylaxis and optimal pre-procedural aseptic measures are recommended before CIED implantation, surgical or transcatheter implantation of a prosthetic valve or other foreign material [16–18]. Prompt therapy with organism sensitive antimicrobial pharmacotherapy should be prescribed for any clinically apparent infection [1].

### Echocardiography

Echocardiography is the pivotal imaging modality in diagnosing suspected IE and should be performed as a first-line screening tool [1]. Echocardiography can assess structural cardiac abnormalities, such as; the presence, number, size, shape, location, echogenicity, and mobility of vegetations. Echocardiography can assess for perivalvular infection (abscess, pseudoaneurysm, new or partial dehiscence of a prosthetic valve), intracardiac fistulae and leaflet prolapse or perforation. Echocardiography can assess for functional abnormalities; the presence and severity of native or prosthetic valve stenosis or regurgitation and ventricular dysfunction [19].

The initial assessment of patients with suspected IE is performed with transthoracic echocardiography (TTE) [20] (Fig. 5A* and *Fig. 7). Its sensitivity to detect vegetations on native valves is about 70%, but drops to 50% in prosthetic valves due to the acoustic shadowing of artificial leaflets [4]. In the early postoperative period, the presence of perivalvular haematoma and oedema may also lead to misinterpretations. TTE is limited in the assessment of perivalvular complications. The sensitivity of TTE for diagnosing abscesses is 30–50%, which increases to 80–90% with transesophageal echocardiography (TOE) [20, 21]. Abscesses are more frequent on prosthetic than native valves and in the aortic than the mitral position. TTE has limited sensitivity (about 25%) to detect small vegetations (< 5 mm) or vegetations associated with intracardiac devices [22].

**Fig. 1:**
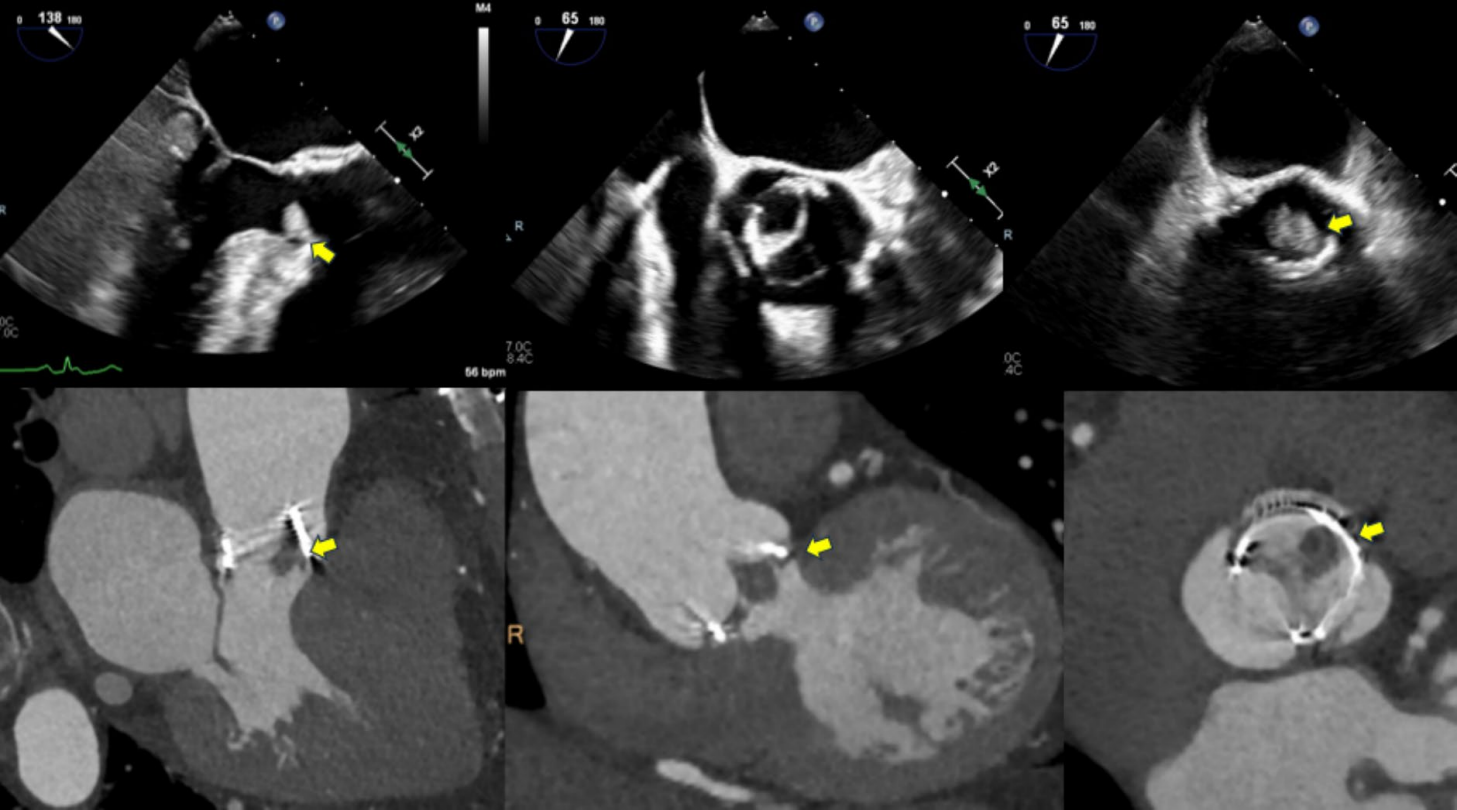
70-year-old female with prior surgical aortic valve replacement, presenting with acute limb ischemia and *Pseudomonas Aeruginosa* bacteremia, raising concern for possible infective endocarditis. Figure 1: *top panel*: transesophageal echocardiogram images with an echodensity on the aortic valve (arrow) suspicious for a vegetation, though the assessment was limited by artifacts. The *bottom panel*: gated cardiac CT imaging, clearly visualizing the vegetation (arrow). CT ruled out aortic root abscess or paravalvular infection. The patient was treated with appropriate antibiotics for infective endocarditis. This case underscores the utility of CT in clarifying equivocal echocardiographic findings in endocarditis management

**Fig. 2:**
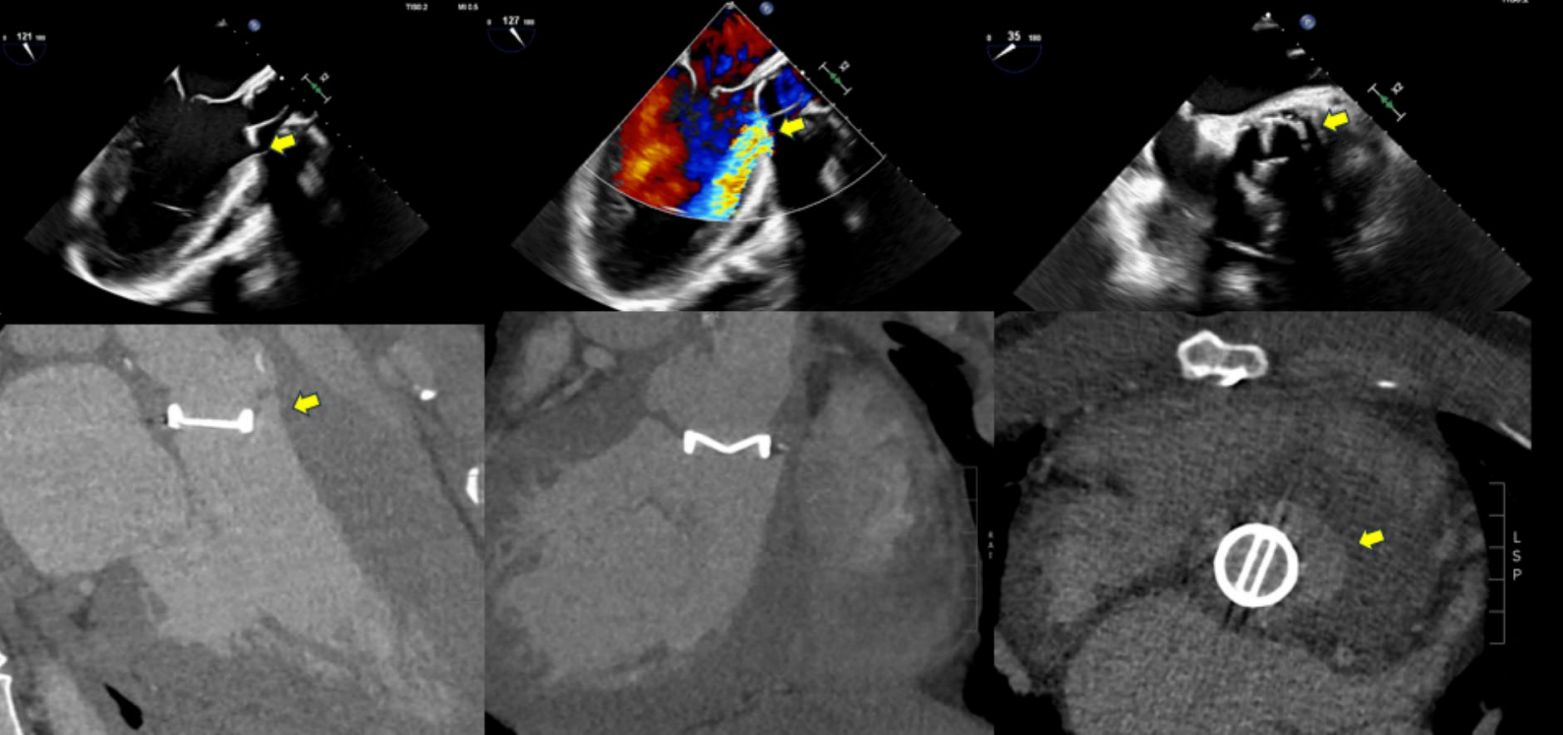
Images of a 44-year-old gentleman with a history of valve-sparing aortic root replacement for an aneurysmal aortic root approximately 9 years ago, followed by mechanical aortic valve replacement (AVR) 5 years later. He presented with heart failure, and his transthoracic echocardiogram revealed severe paravalvular aortic regurgitation. Transesophageal echocardiogram confirmed severe paravalvular regurgitation and valve dehiscence. A subsequent CT coronary angiogram confirmed valve dehiscence and an aortic root abscess which was culture negative. *Top panel:* transesophageal echocardiogram images depicting valve dehiscence and severe paravalvular aortic regurgitation (arrow). *Bottom panel:* CT demonstrating valve dehiscence and the aortic root abscess (arrow). The patient was referred for surgery

**Fig. 3:**
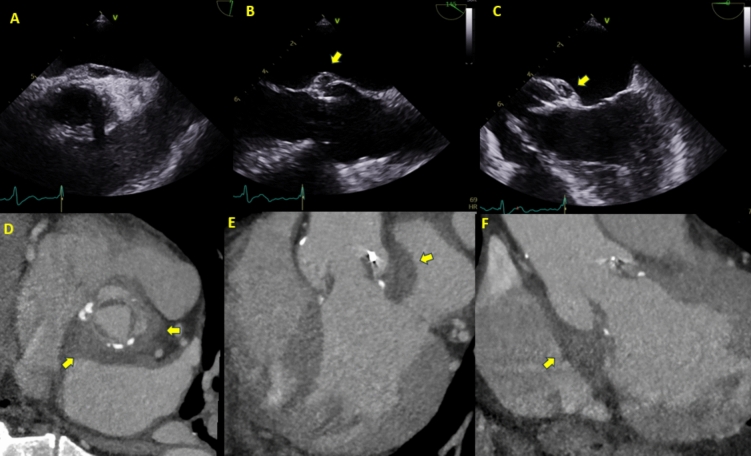
67-year-old gentleman presenting with a 4-day history of malaise, fatigue, and fevers. History of 25 mm Mosaic tissue aortic valve replacement 6 years prior to this presentation. Blood cultures were positive for *Methicillin-Sensitive Staphylococcus Aureus* (MSSA). Transthoracic echocardiogram revealed thickened valve leaflets and abnormal thickening along the posterior aortic root with an echolucent space, suspicious for an aortic root abscess. These findings were confirmed on CT coronary angiogram, which also showed significant thickening of the aortic root, particularly at the non-coronary and left coronary cusp positions. The patient was referred for surgery, during which frank pus was drained from beneath the non-coronary cusp. The *top panel* (A, B, C) shows transesophageal echocardiogram findings suspicious for an aortic root abscess with thickened aortic valve leaflets. The *bottom panel* shows CT coronary angiogram images demonstrating aortic root thickening consistent with the abscess

**Fig. 4:**
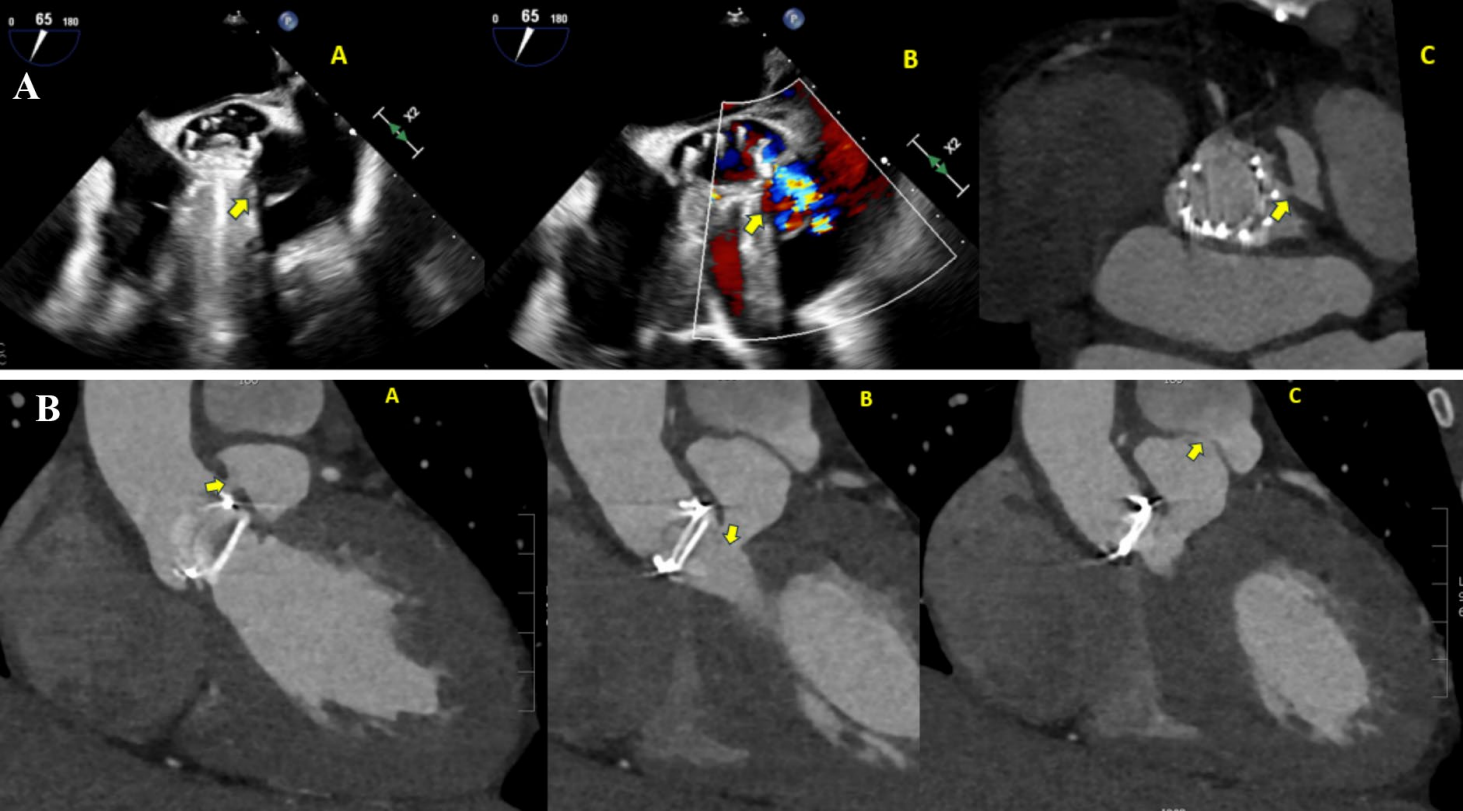
45-year-old female who initially presented with *Enterococcus faecalis* native valve endocarditis, severe aortic regurgitation, and acute heart failure. She underwent emergent mechanical aortic valve replacement (19 mm) and ventricular septal defect (VSD) repair with a bovine pericardial patch. Approximately 6 months later, routine echocardiography (Fig. 4A) revealed abnormal flow and a paravalvular space with communication from the aortic root to the pulmonary artery, raising suspicion for a fistula. CT coronary angiography (Fig. 4B) confirmed an aortic root pseudoaneurysm, which communicated with both the pulmonary artery and the left ventricular outflow tract (LVOT). The patient was referred for re-do surgery. This case highlights the incremental value of CT coronary angiography in identifying aortic root pseudoaneurysm. The pseudoaneurysm was possibly due to reinfection, although the patient was culture-negative at the time of representation

The 2023 European Society of Cardiology (ESC) Guidelines on IE recommend TOE in all patients with suspected IE, due to its higher sensitivity (about 90%) in detecting vegetations and evaluating perivalvular complications (Figs. 1, 2, 3, 4A and 6) relative to transthoracic echocardiography. This recommendation includes those patients with a TTE suggestive of IE, apart from isolated right-sided native valve IE where a good quality and conclusive TTE has been performed [1]. TTE in right sided native valve IE, often provides comprehensive assessment due to the anterior position of the right heart, but sometimes it is necessary to use non-standard echocardiographic views to visualize the right ventricular inflow and outflow tract (Fig. 7), Eustachian valve and posterior leaflet of tricuspid valve [23].

**Fig. 5:**
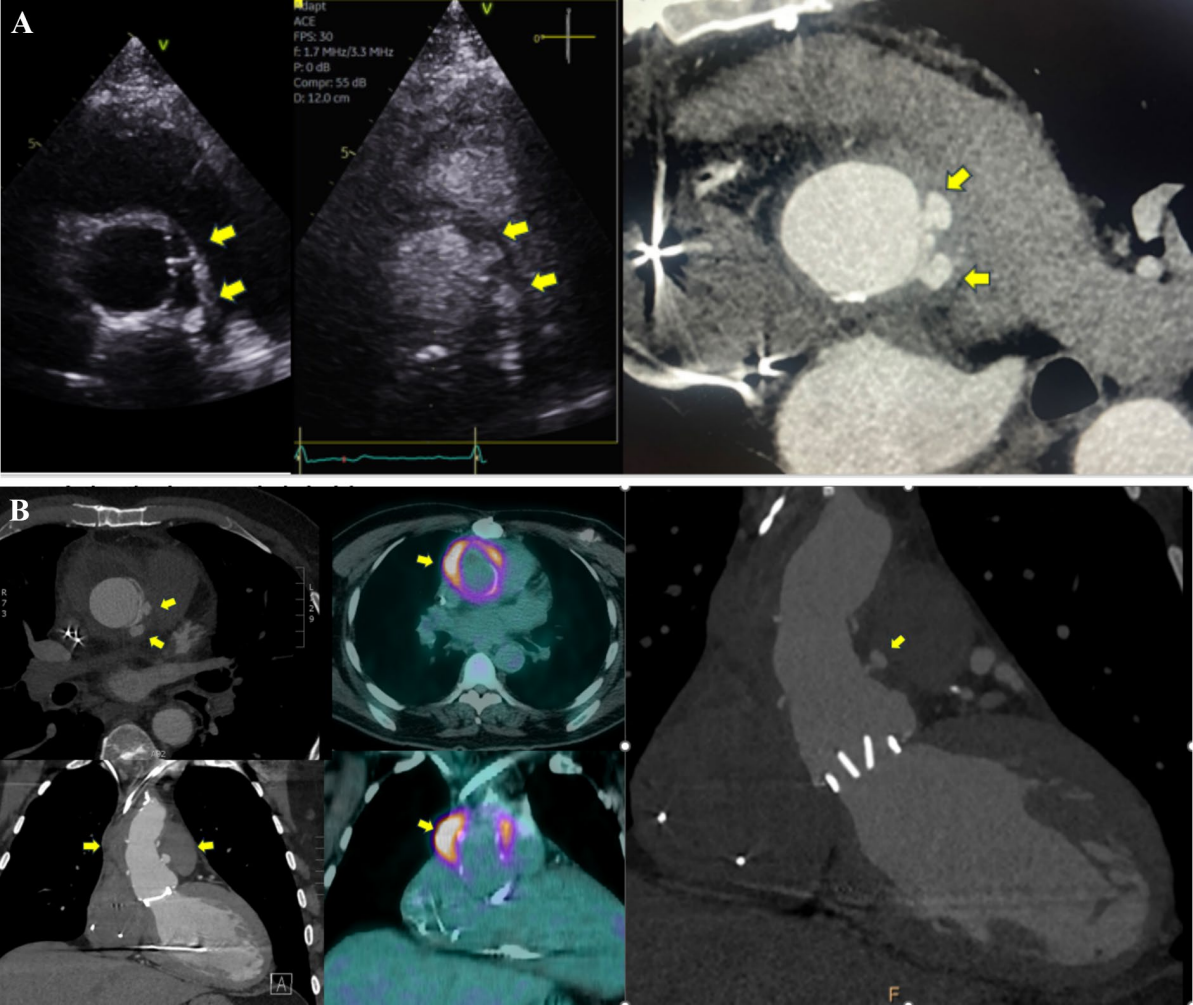
63-year-old gentleman with prior mechanical aortic valve replacement and a supra-coronary ascending aortic graft five years prior, presenting with *Methicillin-Sensitive Staphylococcus Aureus* (MSSA) bacteremia and suspected mechanical aortic valve endocarditis. Transthoracic echocardiogram revealed no vegetation but did show an echolucent space around the ascending aorta graft. Transesophageal echocardiogram could not be performed due to grade D esophagitis and ulcers. The patient was also found to have multifocal cerebritis and small cerebral abscesses on CT brain imaging. A subsequent CT cardiac angiogram revealed a perigraft collection around the ascending aorta graft with two small pseudoaneurysms at the site of the anastomosis between the native aortic root and the supra-coronary ascending aorta graft junction, correlating with the echolucent space seen on echocardiography. FDG-PET imaging confirmed infection with FDG uptake around the graft. Figure 5A shows a side-by-side comparison of the perigraft echolucent space on standard echocardiography, echocardiography with Definity contrast, and CT coronary angiogram (A, B, and C, respectively). Figure 5B reveals the ascending aorta pseudoaneurysm and perigraft collection in both short-axis and long-axis images, as well as FDG uptake on PET imaging in this region. The patient was referred for surgery

**Fig. 6:**
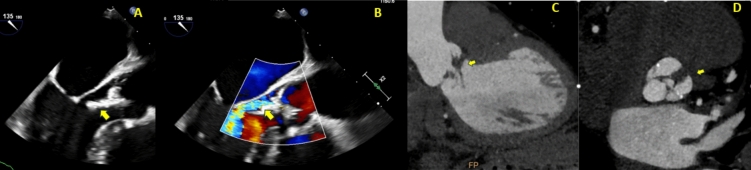
61-year-old gentleman presenting with subacute infective endocarditis. He had a three-week history of night sweats, chills, and rigors, along with a three-to-six-month history of exertional dyspnea and chest tightness. Transesophageal echocardiogram revealed thickening of the aortic valve, mobile vegetations, and moderate aortic regurgitation. A CT coronary angiogram, performed as part of his surgical workup, confirmed thickening of the aortic valve leaflets, a central coaptation defect. *Panel A* shows an abnormal echodensity attached to the aortic valve. *Panel B* shows the aortic regurgitation jet on long-axis images of the transesophageal echocardiogram. *Panel C* shows the filamentous density attached to the thickened aortic valve on CT. *Panel D* reveals the thickened bicuspid aortic valve with a raphe between the left and right coronary cusps and central malcoaptation as the site of the patient's aortic regurgitation

**Fig. 7:**
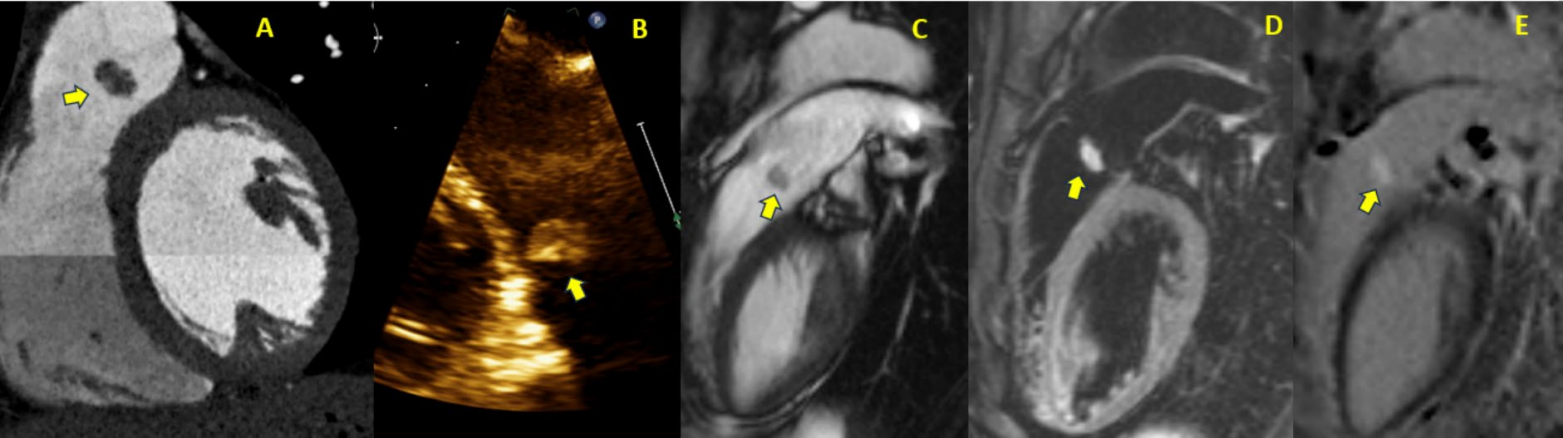
42-year-old female presented with chest pain. CT coronary angiogram, performed to rule out coronary artery disease, incidentally revealed a rounded mass attached to her pulmonic valve leaflet (*Image A*). This finding was confirmed on transthoracic echocardiogram (RVOT view) (*Image B*). The differential diagnosis included a benign tumor or a vegetation, although endocarditis was thought to be less likely as the patient did not have any signs or symptoms of systemic infection. For further tissue characterization, the patient underwent cardiac MRI. SSFP bright blood imaging (*Image C*) showed the mass attached to the pulmonic valve. Edema-weighted imaging (STIR) (*Image D*) revealed increased signal intensity of the pulmonic valve mass, indicating increased water content. Late gadolinium-enhancement imaging (*Image E*) demonstrated diffuse gadolinium uptake within the mass, suggesting a fibroblastoma. This case highlights the tissue characterization capabilities of cardiac MRI using multiple sequences and emphasizes the ability of MRI to differentiate vegetation from a benign tumor, particularly when the echodensity has limited mobility. The patient opted for surgical removal of the mass, which was confirmed to be a pulmonary valve fibroblastoma.

Evaluation with the combination of both TTE and TOE was superior in identifying morphological valve abnormalities, septum defects, and fistula formation compared to other imaging modalities in 46 patients with suspected IE. In addition, echocardiography provides a reliable assessment of ventricular function [24]. Echocardiographic evaluation of vegetation size and mobility is important to predict embolic events and guide the indication and timing of surgery, as well as follow-up during the peri-operative and post-operative period. Several conditions, such as papillary fibroelastoma, myxomatous mitral valve disease or thrombus may be mistaken for a vegetation when assessed with echocardiography (Fig. 7). TOE is the gold standard for vegetation detection and measurement of its length, with 2D-TOE limited in selecting the true maximal dimensions. Real-time three-dimensional TOE (RT3D-TOE) allows for a thorough analysis of vegetation size and morphology and assessment of perivalvular lesions (abscess, valve perforation, prosthetic valve dehiscence) with volumetric reconstruction [25].

In patients with complex congenital heart disease and the presence of prosthetic material, especially in the right heart structures or branch pulmonary arteries, the detection rate of vegetations is low even with the use of TOE [26]. Valve regurgitation may be suggestive of fistula formation or leaflet perforation. A regurgitant jet originating from the central part of a leaflet is highly suggestive of a perforation. In addition, a periprosthetic valve dehiscence with a newly detected regurgitation is highly suspicious of IE (Fig. 2). However, mild isolated periprosthetic regurgitation may also be observed in the absence of infection [27]. The severity of an acute regurgitation should be quantified with both modalities (TTE and TOE) combining anatomical, semiquantitative and quantitative parameters [28].

In case of intracardiac device-related IE, both TTE and TOE have limited diagnostic accuracy. Intracardiac echocardiography (ICE) is an emerging invasive imaging modality that detects device lead abnormalities and small vegetations attached to the leads and is useful to guide transvenous lead extraction and thus minimize the risk for periprocedural complications, such as pulmonary embolization or incomplete removal of vegetations [29].

The timing and echocardiographic mode (TTE or TOE) depend on the initial findings, type of microorganism, and initial response to antibiotics, according to the recent ESC guidelines [1]. In case of a normal or inconclusive TTE or TOE and high clinical suspicion of IE, echocardiography should be repeated within a week. There should be a low threshold for repeating TTE when a new complication is suspected with the option to further evaluate with TOE if required. Repeat TTE should be considered during follow-up of uncomplicated IE, to detect new silent complications and to monitor vegetation size. Echocardiographic evaluation of valve morphology and cardiac function is important at completion of antibiotic therapy or in patients who did not undergo surgery [1].

### CT for IE

CTCA is an effective and readily available method of assessing cardiac valves and the surrounding heart for features of IE. CTCA is most useful when used in conjunction with other forms of imaging and in the presence of prosthetic heart valves where artifact from surgical material can limit the diagnostic performance of other imaging modalities including TTE and TOE (Figs. 1, 2, 3, 4, 5, 6, 7). CTCA also has a role when other forms of imaging are unavailable or contraindicated. CTCA can add valuable information where abscess or pseudoaneurysm formation is suspected. CTCA may aid surgical planning and in some cases, offer an alternative to invasive coronary angiography prior to surgery [30, 31].

A meta-analysis of 8 studies prior to 2019 found a pooled sensitivity of 64% with a specificity of 88% for detection of vegetations on CT. When using multiphase CT sensitivity increased to 86% [30]. More recent studies report a sensitivity of between 80–98.6% of CT for the detection of endocarditis in native valves, with a specificity of 60%−62.5%. In prosthetic valves, a sensitivity of 71.4–81.8% and a specificity of 50% has been reported for endocarditis detection by CT [31, 34].

The size of the vegetation has bearing on the diagnostic performance of CT with smaller vegetations (< 10 mm) having a lower detection rate [32–35]. CTCA reveals the size, location, and attachment characteristics of vegetations with the benefit of high spatial resolution achieved with this modality. Full heartbeat acquisition images can be helpful in assessing the motion of the vegetation.

CTCA is an excellent modality to assess for paravalvular complications of endocarditis (Figs. 1, 3, 4, 5)*.* Particularly where abscess or pseudoaneurysm are suspected [32]. Abscesses appear as paravalvular collections with a characteristic low attenuation. Pseudoaneurysms are irregular shaped cavities in the paravalvular region, these are contiguous with the cardiac or aortic blood pool and may appear pulsatile on full heartbeat acquisition images [36]. Reported sensitivities and specificities for the detection of abscess or pseudoaneurysm by CTCA range from 60 to 100% and 75% to 92% respectively [30, 31]. This is in keeping with the pooled sensitivity (78%) and specificity (92%) for abscess or pseudoaneurysm detection from the meta-analysis from Oliveira et al. [32]. Similar sensitivities of CT in the detection of abscesses and pseudoaneurysms are seen in both native and prosthetic valves demonstrating consistently higher diagnostic yield than TOE in this setting [31, 32, 34].

With adequate image quality CTCA offers a detailed evaluation of valve leaflets, enabling the detection of perforations. Prosthetic valve dehiscence and paravalvular leakage are highly suspicious for IE in the correct clinical context (Fig. 2). Valve dehiscence can be visualized on full heart beat acquisition images by the rocking of the valve with cardiac motion. CTCA can detect dehiscence and leak with a moderate degree of sensitivity ranging from 50 to 88% and a high degree of specificity 100% [31–33].

When assessing for the presence of IE the imaging protocol should be optimised. It is recommended that a low dose non-enhanced cardiac acquisition is followed by an ECG gated, contrast enhanced acquisition. Imaging protocols in the literature range from 30–80% of the RR to 0–100% of the RR acquired in 10 to 25 phase data sets (10% to 4% increments of the RR) [37]. Typical CTCA protocols are useful for assessing left sided valves due to homogenous contrast filling of the LV and LA. Homogenous contrast filling of the RA and RV can be challenging to achieve making right sided valves and pacemaker/ICD leads more challenging to accurately assess. A multiphase injection or a late venous phase are possible ways to optimise right heart imaging [37, 38].

### 18F-fluorodeoxyglucose PET/CT

Nuclear imaging modalities and especially hybrid positron emission tomography/computed tomography (PET/CT) have emerged as useful techniques in the diagnostic work-up of IE especially when echocardiography is suboptimal, or the diagnosis of IE is uncertain [39] (Fig. 5). PET/CT takes advantage of the 18F-fluorodeoxyglucose (^18^F-FDG PET/CT), which is incorporated by accumulated leucocytes, macrophages and CD4 + T-lymphocytes at the sites of infection, providing metabolic information. Using a visual approach, valvular/prosthetic or perivalvular/periprosthetic areas of infection can be appreciated with moderate-high intensity and abnormal patterns of distribution (focal/multifocal or diffuse heterogeneous) [1, 40]. Semiquantitative methods include measurement of the standardized uptake value (SUV). Although there are no specific cut-off values, maximum SUV > 5 (95% CI, 4–15) or SUV ratio (prosthesis-to-background SUV) > 2.5 (95% CI, 2–6) are highly suggestive of infection [1, 41]. Additionally, the administration of intravenous iodinated contrast in the setting of an electrocardiogram (ECG)-gated cardiac CT angiography (PET/CTA ECG-gated cardiac CTA) provides additional anatomic information during the same exam allowing visualisation of IE-related lesions (see CT section). This is particularly helpful in complex settings, such as patients with CHD [39, 42] or aortic grafts [43] (Fig. 5).

This unique ability of providing metabolic information (and anatomical information if contrast is given) has been acknowledged for many years, especially in cases of high clinical suspicion of prosthetic valve endocarditis (PVE), in which cases ^18^F-FDG PET/CT has showed to improve the diagnostic yield compared with the modified Duke Criteria [44]. Interestingly, the incorporation of ^18^F-FDG PET/CT in the 2015 ESC Guidelines for the management of infective endocarditis increased the sensitivity of Duke criteria from 57 to 84% [44]. According to a recent meta-analysis ^18^F-FDG PET/CT has good diagnostic accuracy to detect perivalvular or periprosthetic complications in both native and prosthetic endocarditis and particularly for PVE with 86% sensitivity and 84% specificity [45]. In contrast, in NVE, the sensitivity is low (about 31%) but with a higher specificity (around 98%) [46]. Therefore, the diagnosis of IE cannot be excluded in the absence of [18F]FDG uptake [45, 47]. Nevertheless, PET/CT has demonstrated very high sensitivity and specificity for generator/pocket and extracardiac or intravascular lead infection [48–50].

Another strength of the technique is to identify extracardiac lesions, mycotic aneurysms as well as the primary source of bacteraemia (whole-body ^18^F-FDG PET/CT) [51–55]. While septic emboli are typically located in the spleen, lungs, liver and kidneys, it is not uncommon for other extracardiac manifestations such as spondylodiscitis and septic arthritis to be revealed [51, 56]. However, owing to the high physiological uptake of ^18^F-FDG in the cerebral cortex, this modality does not perform well in the detection of cerebral septic embolism. Lastly, PET/CT can also assess response to therapy and determine the duration of antibiotics, particularly in patients taking long-term antibiotic treatment who have been deemed to be high risk for surgery [41, 50, 57].

Over the years this improved diagnostic performance of the modality led ^18^F-FDG PET/CT to be gradually added into many diagnostic algorithms and guidelines [58], included as a Major Criterion in the 2023 Duke-ISCVID IE Criteria [11] and featuring a central role in the recently published 2023 ESC Guidelines for the management of endocarditis [1]. According to the latter, ^18^F-FDG PET/CT(A) is recommended in suspected PVE in cases of inconclusive echocardiography to confirm the diagnosis (Class I Recommendation, Level of evidence B).

Finally, limitations of ^18^F-FDG PET/CT include restricted access, availability, cost and radiation exposure. To reduce the rate of false positives, patient preparation with a ketogenic diet (high protein, high fat, no carbohydrate) before the exam is of utmost importance [41, 59]. It is important to note the limitations of the technique. Antibiotic treatment can affect metabolism and consequently can impact the results. The presence of surgical adhesives, vasculitis, postsurgical inflammation, neoplasms, active thrombus, atherosclerotic plaque, Libman-Sacks endocarditis may resemble ^18^F-FDG uptake and result in false positives [41, 60].

### WBC SPECT in IE

White Blood Cell Single Photon Emission Computed Tomography (WBC SPECT) scans play a role in the diagnosis and management of infective endocarditis, particularly in cases where echocardiography results are inconclusive, or PET CT is unavailable. This nuclear imaging modality involves the labelling of autologous white blood cells and their subsequent visualization, allowing for the detection of active infection sites with high specificity. WBC SPECT has demonstrated utility in identifying prosthetic valve endocarditis and differentiating infectious from non-infectious causes of valvular lesions, thereby guiding clinical decision-making and antimicrobial therapy. The sensitivity has been reported as between 64–90% and the specificity 36–100% with diagnostic yield increasing in suspected cases of periprosthetic abscesses. The significant disadvantages of this modality include its time-consuming nature, centre specific availability and elevated false negative rate, particularly in patients treated with antibiotics. Recent studies have shown that WBC SPECT offers superior diagnostic accuracy compared to traditional imaging techniques such as echocardiography alone, especially in complex or prosthetic cases and in cases of possible intracardiac device infection [61, 62]. The 2023 ESC guidelines on endocarditis diagnosis recommend WBC SPECT as an alternative to PET-CT for detecting prosthetic valve endocarditis, underscoring its clinical value [1]. However, WBC SPECT is not incorporated into the modified Duke criteria for the diagnosis of infective endocarditis in 2023, which primarily rely on clinical, microbiological, and echocardiographic findings [10]. Incorporating WBC SPECT into diagnostic algorithms can enhance detection accuracy and aid treatment monitoring, ultimately improving patient outcomes.

### Cardiac MRI in IE

The role of cardiac MRI in the diagnosis and management of infective endocarditis has not yet been included in clinical guidelines [1]. Cardiac MRI is limited by spatial resolution (as compared with cardiac CT) and signal void generated by prostheses impairing the assessment of prosthetic valve anatomy and function [1].

The potential diagnostic value of cardiac MRI is the ability to apply tissue characterization to help differentiate between potential alternative diagnoses such as myxomas, papillary fibroelastomas, lipomas, thrombi, and metastasis and to provide a non-invasive multiplanar assessment regarding the precise location in relation to other structures [63–66] (Fig. 7).

SSFP (steady state free precession) images in Cine mode can identify the number and size of valvular vegetations with varying degrees of success [8, 63]. Flow haemodynamic assessment with cardiac MRI can provide quantitative assessment of valvular regurgitant and stenotic lesions as well as providing the gold standard in the assessment of ventricular volumes and systolic function. Such quantitative measures are highly accurate, precise, and reproducible across studies [64].

Early and late gadolinium contrast enhanced imaging can assess the endothelial lining for direct and flow-related extension of tissue inflammation in the diagnosis of infective endocarditis [9]. Delayed enhancement of the endothelial lining may facilitate early diagnosis of endocarditis by detecting retrograde or antegrade dissemination of infection with contrast enhancement even in the absence of a discrete vegetation on TTE and MRI cine imaging [9]. Rest perfusion imaging can provide information regarding vascularization and in combination with early gadolinium enhancement (EGE) can assess for the presence of thrombus. Evaluation of morphologic T1 weighted and T2 weighted imaging and fat suppression imaging, as well as T1 and T2 quantitative mapping may assist in excluding other potential differential diagnoses such as benign and malignant cardiac tumours [63–66] (Fig. 7).

Cardiac MRI may detect complications of endocarditis such as paravalvular extension of infection prior to the formation of abscess, mycotic aneurysm or intracardial fistula. It may detect or exclude myocardial infarction and fibrosis in the setting of vegetations causing coronary artery embolization, myocarditis or pericarditis, perforation, endocardial jet lesions or intracardiac shunting [8, 63–66].

Like all investigations, cardiac MRI has both advantages and limitations when compared to other imaging modalities. Cardiac MRI may add useful diagnostic information when used as part of a multimodality imaging assessment in the cases where intracardiac tumour or thrombus are potential alternative diagnoses. Metallic artifacts secondary to prosthetic valves or implantable cardiac devices may limit the diagnostic utility. The availability and expense as well as the longer acquisition times must be considered, and it requires expertise in protocolling and acquisition to produce diagnostically useful images. Cardiac MRI avoids exposure to ionizing radiation which is associated with CT and PET imaging [63–66].

## Conclusion

The diagnosis of infective endocarditis is an evolving landscape. There is scope within our practice as clinicians to improve patient education particularly amongst those who are at the highest risk of endocarditis to facilitate prevention and earlier detection of a condition where the mortality remains high. Due to the high morbidity and mortality prevention strategies were one of the focus points of the recent ESC guidelines [1]. Multimodality imaging provides a set of tools which when used in combination, add incremental value and in many cases can improve the timely diagnosis of infective endocarditis and identify its complications and progress with therapy. A patient centred approach utilising local, available, site-specific multi-modality imaging expertise should be considered. A combination of cardiac CT, FDG-PET CT, WBC SPECT should be considered, and in specific cases the addition of cardiac MRI can complement the well-established and validated role of transthoracic and transoesophageal echocardiographic imaging in ensuring timely diagnosis and assessment for life threatening complications, which may ultimately improve patient outcomes.

## Data Availability

No datasets were generated or analysed during the current study.

## Abbreviations

CT: Computerised tomography
MRI: Magnetic resonance imaging
PET: Positron emission tomography
IE: Infective endocarditis
^18^F-FDG PET/CT: 18F-fluorodeoxyglucose positron emission tomography/computed tomography
PVE: Prosthetic valve endocarditis
NVIE: Native valve infective endocarditis
WBC SPECT: White blood cell single-photon emission computed tomography
